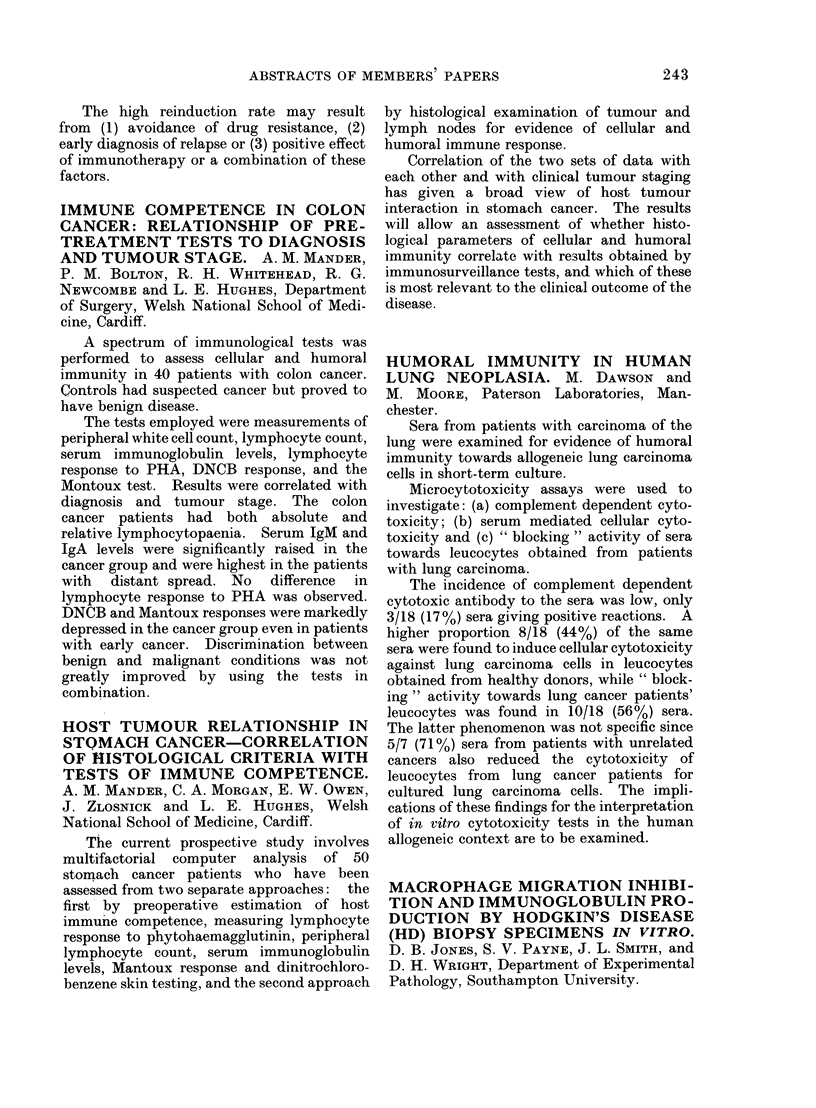# Proceedings: Humoral immunity in human lung neoplasms.

**DOI:** 10.1038/bjc.1975.166

**Published:** 1975-08

**Authors:** M. Dawson, M. Moore


### HUMORAL IMMUNITY IN HUMAN LUNG NEOPLASIA.

M. Dawson and M. Moore, Paterson Laboratories, Manchester.

Sera from patients with carcinoma of the lung were examined for evidence of humoral immunity towards allogeneic lung carcinoma cells in short-term culture.

Microcytotoxicity assays were used to investigate: (a) complement dependent cytotoxicity; (b) serum mediated cellular cytotoxicity and (c) " blocking " activity of sera towards leucocytes obtained from patients with lung carcinoma.

The incidence of complement dependent cytotoxic antibody to the sera was low, only 3/18 (17%) sera giving positive reactions. A higher proportion 8/18 (44%) of the same sera were found to induce cellular cytotoxicity against lung carcinoma cells in leucocytes obtained from healthy donors, while " blocking " activity towards lung cancer patients' leucocytes was found in 10/18 (56%) sera. The latter phenomenon was not specific since 5/7 (71%) sera from patients with unrelated cancers also reduced the cytotoxicity of leucocytes from lung cancer patients for cultured lung carcinoma cells. The implications of these findings for the interpretation of *in vitro* cytotoxicity tests in the human allogeneic context are to be examined.